# Supplementary material for: Short-range interactions between fibrocytes and CD8+ T cells in COPD bronchial inflammatory response
Source: eLife. 2023 Jul 26;12:RP85875. doi: 10.7554/eLife.85875 (PMC10371228; doi:10.7554/eLife.85875)
Supplement: Supplementary file 1. — FEV1, forced expiratory volume in 1 s; FVC, forced vital capacity; LFT, lung function test; RV, residual volume; TLCO, Transfer Lung capacity of Carbon monoxide, PaO2, partial arterial oxygen pressure, PaCO2, partial arterial carbon dioxide pressure; WA, mean wall area; LA, mean lumen area, WA%, mean wall area percentage; WT, wall thickness; LAA, low-attenuation area; MLA E or I, mean lung attenuation value during expiration or inspiration. MLA I-E, the difference between inspiratory and expiratory mean lung attenuation value. %CSA<5, percentage of total lung area taken up by the cross-sectional area of pulmonary vessels less than 5 mm2; %CSA5–10, percentage of total lung area taken up by the cross-sectional area of pulmonary vessels between 5 and 10 mm2; CSN<5, number of vessels less than 5 mm2 normalized by total lung area; CSN5-10, number of vessels between 5 and 10 mm2 normalized by total lung area; NR: not relevant. The correlation coefficient (r), 95% confidence interval, and significance level (p value), were obtained by using nonparametric Spearman analysis. [file elife-85875-supp1.docx]

**Supplementary file 1. Association between density of fibrocytes and clinical characteristics**

|  |  |  | **Density of fibrocytes** | | | |
| --- | --- | --- | --- | --- | --- | --- |
|  |  | **Spearman r** | | **95% confidence interval** | **P value** | |
| Age (yrs.) | | -0,14 | | [ -0.48 to 0.23] | 0,43 | |
| Body-mass index (kg/m^2^)  Pack years (no.)  **LFT**  FEV_1_ (% pred.)  FEV_1_/FVC ratio (%)  FVC (% pred.)  RV (% pred.)  TLCO (% pred.)  **Six-minute walk test distance (m)**  **Arterial blood gases**  PaO_2_ (mm Hg)  PaCO_2_ (mm Hg)  **CT parameters**  Bronchi:  WA4%  WT4 (mm)  WA5%  WT5 (mm)  Emphysema:  LAA (%)  Air trapping:  MLA E (HU)  MLA I (HU)  MLA I-E (HU)  Pulmonary Vessels  %CSA_<5_  %CSA_5-10_  CSN_<5_  CSN_5-10_ | | -0,12  0,14  -0,44  -0,38  -0,38  0,30  -0,56  -0,14  0,08  0,26  0,08  0,03  0,10  0,19  0,40  -0,38  -0,36  -0,15  -0,41  -0,22  -0,38  -0,41 | | [ -0.46 to 0.25]  [ -0.24 to 0.48]  [-0.69 to -0.10]  [-0.65 to -0.03]  [-0.65 to -0.03]  [-0.07 to 0.59]  [-0.78 to -0.24]  [-0.52 to 0.29]  [-0.30 to 0.44]  [-0.13 to 0.57]  [-0.33 to 0.46]  [-0.37 to 0.42]  [-0.31 to 0.48]  [-0.23 to 0.55]  [0.04 to 0.67]  [-0.69 to 0.04]  [-0.65 to 0.02]  [-0.53 to 0.28]  [-0.68 to -0.05]  [-0.55 to 0.16]  [-0.66 to -0.02]  [-0.68 to -0.05] | 0,51  0,46  **0,01**  **0,03**  **0,03**  0,10  **0,001**  0,52  0,67  0,17  0,71  0,88  0,62  0,36  **0,03**  0,07  0,053  0,48  **0,02**  0,24  **0,04**  **0,02** | |
|  | |  |  | | |  |
