# Supplementary material for: Short-range interactions between fibrocytes and CD8+ T cells in COPD bronchial inflammatory response
Source: eLife. 2023 Jul 26;12:RP85875. doi: 10.7554/eLife.85875 (PMC10371228; doi:10.7554/eLife.85875)
Supplement: Supplementary file 2. — FEV1, forced expiratory volume in 1 second; FVC, forced vital capacity; LFT, lung function test; RV, residual volume; TLCO, Transfer Lung capacity of Carbon monoxide, PaO2, partial arterial oxygen pressure, PaCO2, partial arterial carbon dioxide pressure; WA, mean wall area; LA, mean lumen area, WA%, mean wall area percentage; WT, wall thickness; LAA, low-attenuation area; MLA E or I, mean lung attenuation value during expiration or inspiration. MLA I-E, the difference between inspiratory and expiratory mean lung attenuation value. %CSA<5, percentage of total lung area taken up by the cross-sectional area of pulmonary vessels less than 5 mm2; %CSA5–10, percentage of total lung area taken up by the cross-sectional area of pulmonary vessels between 5 and 10 mm2; CSN<5, number of vessels less than 5 mm2 normalized by total lung area; CSN5-10, number of vessels between 5 and 10 mm2 normalized by total lung area; NR: not relevant. The correlation coefficient (r), 95% confidence interval, and significance level (p value), were obtained by using nonparametric Spearman analysis. [file elife-85875-supp2.docx]

**Supplementary file 2. Association between density of CD8^+^ T cells and clinical characteristics**

|  |  |  | **Density of CD8^+^ T cells** | | | |
| --- | --- | --- | --- | --- | --- | --- |
|  |  | **Spearman r** | | **95% confidence interval** | **P value** | |
| Age (yrs.) | | -0,09 | | [-0.43 to 0.28] | 0,63 | |
| Body-mass index (kg/m^2^)  Pack years (no.)  **LFT**  FEV_1_ (% pred.)  FEV_1_/FVC ratio (%)  FVC (% pred.)  RV (% pred.)  TLCO (% pred.)  **Six-minute walk test distance (m)**  **Arterial blood gases**  PaO_2_ (mm Hg)  PaCO_2_ (mm Hg)  **CT parameters**  Bronchi:  WA4%  WT4 (mm)  WA5%  WT5 (mm)  Emphysema:  LAA (%)  Air trapping:  MLA E (HU)  MLA I (HU)  MLA I-E (HU)  Pulmonary Vessels  %CSA_<5_  %CSA_5-10_  CSN_<5_  CSN_5-10_ | | -0,07  0,33  -0,25  -0,30  -0,18  0,24  -0,25  -0,06  0,02  0,40  0,35  0,31  0,32  0,31  0,39  -0,49  -0,48  0,15  -0,38  -0,44  -0,29  -0,41 | | [-0.42 to 0.30]  [-0.04 to 0.62]  [ -0.56 to 0.12]  [ -0.60 to 0.06]  [ -0.50 to 0.19]  [ -0.13 to 0.55]  [ -0.57 to 0.14]  [ -0.46 to 0.37]  [-0.35 to 0.39]  [ 0.04 to 0.67]  [ -0.05 to 0.66]  [ -0.09 to 0.63]  [ -0.09 to 0.64]  [ -0.10 to 0.63]  [ 0.02 to 0.66]  [ -0.75 to -0.09]  [ -0.73 to -0.13]  [ -0.28 to 0.53]  [ -0.66 to -0.01]  [ -0.70 to -0.08]  [ -0.59 to 0.09]  [ -0.68 to -0.05] | 0,71  0,07  0,16  0,09  0,33  0,19  0,19  0,79  0,91  **0,03**  0,08  0,12  0,11  0,12  **0,03**  **0,02**  **0,008**  0,48  **0,04**  **0,02**  0,12  **0,02** | |
|  | |  |  | | |  |
