# Supplementary material for: Short-range interactions between fibrocytes and CD8+ T cells in COPD bronchial inflammatory response
Source: eLife. 2023 Jul 26;12:RP85875. doi: 10.7554/eLife.85875 (PMC10371228; doi:10.7554/eLife.85875)
Supplement: Supplementary file 3. — FEV1, forced expiratory volume in 1 s; FVC, forced vital capacity; LFT, lung function test; RV, residual volume; TLCO, Transfer Lung capacity of Carbon monoxide, PaO2, partial arterial oxygen pressure, PaCO2, partial arterial carbon dioxide pressure; WA, mean wall area; LA, mean lumen area, WA%, mean wall area percentage; WT, wall thickness; LAA, low-attenuation area; MLA E or I, mean lung attenuation value during expiration or inspiration. MLA I-E, the difference between inspiratory and expiratory mean lung attenuation value. %CSA<5, percentage of total lung area taken up by the cross-sectional area of pulmonary vessels less than 5 mm2; %CSA5–10, percentage of total lung area taken up by the cross-sectional area of pulmonary vessels between 5 and 10 mm2; CSN<5, number of vessels less than 5 mm2 normalized by total lung area; CSN5-10, number of vessels between 5 and 10 mm2 normalized by total lung area; NR: not relevant. The correlation coefficient (r), 95% confidence interval, and significance level (p value), were obtained by using nonparametric Spearman analysis. [file elife-85875-supp3.docx]

**Supplementary file 3. Association between interacting cell density and clinical characteristics**

|  |  |  | **Interacting cells density (D8)** | | | |
| --- | --- | --- | --- | --- | --- | --- |
|  |  | **Spearman r** | | **95% confidence interval** | **P value** | |
| Age (yrs.) | | -0,17 | | [-0.50 to 0.20] | 0,34 | |
| Body-mass index (kg/m^2^)  Pack years (no.)  **LFT**  FEV_1_ (% pred.)  FEV_1_/FVC ratio (%)  FVC (% pred.)  RV (% pred.)  TLCO (% pred.)  **Six-minute walk test distance (m)**  **Arterial blood gases**  PaO_2_ (mm Hg)  PaCO_2_ (mm Hg)  **CT parameters**  Bronchi:  WA4%  WT4 (mm)  WA5%  WT5 (mm)  Emphysema:  LAA (%)  Air trapping:  MLA E (HU)  MLA I (HU)  MLA I-E (HU)  Pulmonary Vessels  %CSA_<5_  %CSA_5-10_  CSN_<5_  CSN_5-10_ | | -0,13  0,22  -0,38  -0,37  -0,27  0,15  -0,50  -0,25  0,25  0,36  0,04  0,02  0,11  0,17  0,30  -0,39  -0,38  0,02  -0,39  -0,22  -0,32  -0,39 | | [-0.47 to 0.24]  [-0.16 to 0.55]  [-0.65 to -0.02]  [-0.64 to -0.02]  [-0.57 to 0.10]  [-0.22 to 0.48]  [-0.74 to -0.16]  [-0.60 to 0.18]  [-0.13 to 0.57]  [-0.01 to 0.64]  [-0.36 to 0.43]  [-0.38 to 0.41]  [-0.30 to 0.49]  [-0.24 to 0.53]  [-0.08 to 0.60]  [-0.69 to 0.026]  [-0.66 to 0.001]  [-0.40 to 0.43]  [-0.66 to -0.02]  [-0.54 to 0.16]  [-0.61 to 0.06]  [-0.66 to -0.02] | 0,48  0,23  **0,03**  **0,04**  0,13  0,41  **0,005**  0,24  0,18  0,05  0,83  0,93  0,58  0,41  0,11  0,06  **0,04**  0,92  **0,03**  0,24  0,09  **0,03** | |
|  | |  |  | | |  |
