# Supplementary material for: Short-range interactions between fibrocytes and CD8+ T cells in COPD bronchial inflammatory response
Source: eLife. 2023 Jul 26;12:RP85875. doi: 10.7554/eLife.85875 (PMC10371228; doi:10.7554/eLife.85875)
Supplement: Supplementary file 4. — FEV1, forced expiratory volume in 1 s; FVC, forced vital capacity; LFT, lung function test; RV, residual volume; TLCO, Transfer Lung capacity of Carbon monoxide, PaO2, partial arterial oxygen pressure, PaCO2, partial arterial carbon dioxide pressure; WA, mean wall area; LA, mean lumen area, WA%, mean wall area percentage; WT, wall thickness; LAA, low-attenuation area; MLA E or I, mean lung attenuation value during expiration or inspiration. MLA I-E, the difference between inspiratory and expiratory mean lung attenuation value. %CSA<5, percentage of total lung area taken up by the cross-sectional area of pulmonary vessels less than 5 mm2; %CSA5–10, percentage of total lung area taken up by the cross-sectional area of pulmonary vessels between 5 and 10 mm2; CSN<5, number of vessels less than 5 mm2 normalized by total lung area; CSN5-10, number of vessels between 5 and 10 mm2 normalized by total lung area; NR: not relevant. The correlation coefficient (r), 95% confidence interval, and significance level (p value), were obtained by using nonparametric Spearman analysis. [file elife-85875-supp4.docx]

**Table S4. Association between the mean minimal distance between fibrocytes and CD8^+^ T cells and clinical characteristics**

|  |  |  | **Mean minimal distance between fibrocytes and CD8^+^ T cells** | | | |
| --- | --- | --- | --- | --- | --- | --- |
|  |  | **Spearman r** | | **95% confidence interval** | **P value** | |
| Age (yrs.) | | -0,02 | | [-0.38 to 0.35] | 0,92 | |
| Body-mass index (kg/m^2^)  Pack years (no.)  **LFT**  FEV_1_ (% pred.)  FEV_1_/FVC ratio (%)  FVC (% pred.)  RV (% pred.)  TLCO (% pred.)  **Six-minute walk test distance (m)**  **Arterial blood gases**  PaO_2_ (mm Hg)  PaCO_2_ (mm Hg)  **CT parameters**  Bronchi:  WA4%  WT4 (mm)  WA5%  WT5 (mm)  Emphysema:  LAA (%)  Air trapping:  MLA E (HU)  MLA I (HU)  MLA I-E (HU)  Pulmonary Vessels  %CSA_<5_  %CSA_5-10_  CSN_<5_  CSN_5-10_ | | -0,10  -0,10  0,24  0,42  0,10  -0,14  0,27  -0,02  -0,05  -0,45  -0,31  -0,24  -0,52  -0,50  -0,36  0,52  0,50  -0,19  0,19  0,29  0,15  0,26 | | [-0.45 to 0.27]  [-0.46 to 0.29]  [-0.13 to 0.56]  [0.06 to 0.68]  [-0.27 to 0.45]  [-0.48 to 0.24]  [-0.12 to 0.59]  [-0.44 to 0.41]  [-0.42 to 0.33]  [-0.70 to -0.09]  [-0.64 to 0.11]  [-0.59 to 0.18]  [-0.76 to -0.14]  [-0.75 to -0.12]  [-0.65 to 0.02]  [0.12 to 0.77]  [0.15 to 0.74]  [-0.57 to 0.25]  [-0.20 to 0.53]  [-0.10 to 0.60]  [-0.24 to 0.59]  [-0.14 to 0.57) | 0,58  0,62  0,19  **0,02**  0,59  0,45  0,16  0,92  0,79  **0,01**  0,13  0,24  **0,008**  **0,01**  0,06  **0,01**  **0,006**  0,38  0,32  0,13  0,43  0,20 | |
|  | |  |  | | |  |
