# Supplementary material for: Short-range interactions between fibrocytes and CD8+ T cells in COPD bronchial inflammatory response
Source: eLife. 2023 Jul 26;12:RP85875. doi: 10.7554/eLife.85875 (PMC10371228; doi:10.7554/eLife.85875)
Supplement: Supplementary file 6. — FEV1, forced expiratory volume in 1 s; FVC, forced vital capacity. [file elife-85875-supp6.docx]

**Supplementary file 6. Multivariate analysis of FEV_1_/FVC**

| **FEV_1_/FVC** | | | | | |
| --- | --- | --- | --- | --- | --- |
| **Model** | **Explicative**  **variables** | **Coefficient** | **Standard error** | **T-value** | **P value** |
| R^2^= 0.39,  Adjusted R^2^= 0.35  P = 0.0009  F = 9.04  Residual standard error = 0.12 | Interacting cells density  Density of mixed cells clusters | -0.0025  0.00230 | 0.0006  0.0011 | -4.0  2.2 | 0.0004  0.04 |
