# Supplementary material for: Short-range interactions between fibrocytes and CD8+ T cells in COPD bronchial inflammatory response
Source: eLife. 2023 Jul 26;12:RP85875. doi: 10.7554/eLife.85875 (PMC10371228; doi:10.7554/eLife.85875)
Supplement: Supplementary file 7. — Plus–minus values are means ± SD. PFT, pulmonary function test; FEV1, forced expiratory volume in 1 s; FVC, forced vital capacity. [file elife-85875-supp7.docx]

**Supplementary file 7. Patient characteristics (for tissular CD8^+^ T cells purification)**

|  |  | **COPD** | **Control** |
| --- | --- | --- | --- |
| n | | 20 | 26 |
| Age (yr) | | 64.0 ± 9.5 | 65.7 ± 12.5 |
| Sex (Men/Woman) | | 12/8 | 11/15 |
| Body-mass index (kg/m^2^)  Current smoker (Y/N)  Former smoker (Y/N)  Pack years (no.)  **PFT**  FEV_1_ (% pred.)  FEV_1_/FVC ratio (%) | | 23.8 ± 4.0  6/14  14/6  48.2 ± 26.9  60.7 ± 27.0  55.1 ± 13.7 | 25.9 ± 4.2  0/26  19/7  24.0 ± 21.7  98.1 ± 20.2  79.3 ± 6.4 |
