# Supplementary material for: Short-range interactions between fibrocytes and CD8+ T cells in COPD bronchial inflammatory response
Source: eLife. 2023 Jul 26;12:RP85875. doi: 10.7554/eLife.85875 (PMC10371228; doi:10.7554/eLife.85875)
Supplement: Supplementary file 8. — Plus–minus values are means ± SD. PFT, pulmonary function test; FEV1, forced expiratory volume in 1 s; FVC, forced vital capacity; PaO2, partial arterial oxygen pressure, PaCO2, partial arterial carbon dioxide pressure. [file elife-85875-supp8.docx]

**Supplementary file 8: Patient characteristics (for circulating CD8^+^/CD4^+^ T cells and fibrocyte precursors purification)**

|  |  | **COPD** |
| --- | --- | --- |
| n | | 44 |
| Age (years) | | 67.4 ± 7.7 |
| Sex (Men/Woman) | | 24/20 |
| Body-mass index (kg/m^2^)  Current /Former smokers  Pack years (no.)  **PFT**  FEV_1_ (% pred.)  FEV_1_/FVC ratio (%)  FVC (% pred.)  **Six-minute walk test distance (m)**  **Arterial blood gases**  PaO_2_ (mm Hg)  PaCO_2_ (mm Hg) | | 29.2 ± 7.0  16/28  48.1 ± 19.3  59.7 ± 18.0  56.9 ± 11.5  83.3 ± 15.4  450 ± 133  73.7 ± 11.2  38.8 ± 5.3 |
