# Supplementary material for: Short-range interactions between fibrocytes and CD8+ T cells in COPD bronchial inflammatory response
Source: eLife. 2023 Jul 26;12:RP85875. doi: 10.7554/eLife.85875 (PMC10371228; doi:10.7554/eLife.85875)
Supplement: Supplementary file 9. — Plus–minus values are means ± SD. PFT, pulmonary function test; FEV1, forced expiratory volume in 1 s; FVC, forced vital capacity. [file elife-85875-supp9.docx]

**Supplementary file 9: Patient characteristics (for basal bronchial epithelial cell purification)**

|  |  | **Patients** |
| --- | --- | --- |
| n | | 2 |
| Age (yr) | | 64.1 ± 9.6 |
| Sex (Men/Woman) | | 0/2 |
| Body-mass index (kg/m^2^)  Current smoker (Y/N)  Former smoker (Y/N)  Pack years (no.)  **PFT**  FEV_1_ (% pred.)  FEV_1_/FVC ratio (%) | | 19.0 ± 3.4  1/1  1/1  22.5 ± 24.7  90.4 ± 20.3  70.0 ± 7.7 |
