# Supplementary material for: Short-range interactions between fibrocytes and CD8+ T cells in COPD bronchial inflammatory response
Source: eLife. 2023 Jul 26;12:RP85875. doi: 10.7554/eLife.85875 (PMC10371228; doi:10.7554/eLife.85875)
Supplement: Supplementary file 10. [file elife-85875-supp10.docx]

**Supplementary file 10. Definition of the notations and parameters of the mathematical model**

|  | **Symbol** | **Meaning** |
| --- | --- | --- |
| **General** | $L$ | Lamina propria (=peribronchial area) |
|  | $x_{0}$ | Side length of the units of the lattice $L$ |
|  | $M(s)$ | Neighbourhood of the site $(s)$ |
|  | $V(s)$ | Number of F and C cells belonging to $M(s)$ |
|  | $V\left( F \right)\left( s \right)$ | Number of F cells belonging to $M(s)$ |
|  | $V(C)\left( s \right)$ | Number of C cells belonging to $M(s)$ |
|  | $N_{k}\left( F \right)$ | Number of F cells at the beginning of period $k$ |
|  | $N_{k}\left( C \right)$ | Number of C cells at the beginning of period $k$ |
| **Initial situation** | $n_{0}\left( C \right)$ | Initial density of C cells |
|  | $n_{0}\left( F \right)$ | Initial density of F cells |
|  | $N_{0}\left( C \right)$ | Initial number of C cells |
|  | $N_{0}\left( F \right)$ | Initial number of F cells |
| **Cell death** | $p_{dF}$ | Probability for a F cell to die |
|  | $p_{dC}$ | Basal probability for a C cell to die |
|  | $p_{dC+}$ | Increased probability for a C cell to die |
|  | σ | Threshold number of neighbouring C cells, above which the probability of dying is increased from $p_{dC}$ to $p_{dC+}$ |
| **Cell proliferation** | $p_{F}$ | Probability for a F cell to divide |
|  | $p_{C}$ | Basal probability for a C cell to divide |
|  | $p_{C/F}$ | Increased probability for a C cell to divide |
|  | λ | Threshold number of neighbouring C cells of an empty s’ site belonging to M(s), above which the considered C cell does not divide. |
| **Cell displacement** | $P_{F}(s, s^{'})$ | Probability for a F cell to go from s to s’ |
|  | $P_{C}(s, s^{'})$ | Probability for a C cell to go from s to s’ |
|  | $f_{F}$ | Function partially defining $P_{F}(s, s^{'})$ when $s^{'}\in M\left( s \right), s^{'} is empty and s'\neq s$, depending on $V(C)\left( s' \right)$ |
|  | $f_{C}$ | Function partially defining $P_{C}(s, s^{'})$ when $s^{'}\in M\left( s \right), s^{'} is empty and s'\neq s$, depending on $V\left( s' \right)$ |
|  | $\varepsilon_{F}$ | Value taken by $f_{F}$to reflect a low attraction |
|  | $\varepsilon_{C}$ | Value taken by $f_{C}$to reflect a low attraction |
| **Cell infiltration** | $p_{istaF}$ | Probability for a F cell to get infiltrated at the beginning of a 3 minutes-period |
|  | $p_{istaC}$ | Probability for a C cell to get infiltrated at the beginning of a 3 minutes-period |
|  | $p_{iexaF}$ | Probability for a F cell to get infiltrated during an exacerbation |
|  | $p_{iexaC}$ | Probability for a F cell to get infiltrated during an exacerbation |
|  | $N_{iexaF}$ | Number of F cells that are infiltrated during an exacerbation |
|  | $N_{iexaC}$ | Number of C cells that are infiltrated during an exacerbation |
