# Supplementary material for: Short-range interactions between fibrocytes and CD8+ T cells in COPD bronchial inflammatory response
Source: eLife. 2023 Jul 26;12:RP85875. doi: 10.7554/eLife.85875 (PMC10371228; doi:10.7554/eLife.85875)
Supplement: Supplementary file 11. [file elife-85875-supp11.docx]

**Supplementary file 11**. Numerical values of parameters depending in control and COPD situations

| **Symbol** | **Numerical values** | |
| --- | --- | --- |
|  | **Control** | **COPD** |
| $x_{0}$ | 7 µm | |
| $N_{0}\left( C \right)$ | 118 cells | |
| $N_{0}\left( F \right)$ | 19 cells | |
| $p_{dF}$ | 4.8.10^-6^ | 2.4.10^-6^ |
| $p_{dC}$ | 1.10^-4^ | 5.10^-5^ |
| $p_{dC+}$ | 4.10^-4^ | 2.10^-4^ |
| σ | 3 | |
| $p_{F}$ | 0 | |
| $p_{C}$ | 5.10^-5^ | |
| $p_{C/F}$ | 2.10^-4^ | |
| λ | 3 | |
| $\varepsilon_{F}$ | 10^-3^ | |
| $\varepsilon_{C}$ | 10^-3^ | |
| $p_{istaF}$ | 9.12.10^-5^ | |
| $p_{istaC}$ | 1.40.10^-2^ | |
| $p_{iexaF}$ | 0 | 2.20.10^-3^ |
| $p_{iexaC}$ | 0 | |
| $N_{iexaF}$ | 0 | 1 |
| $N_{iexaC}$ | 0 | |
